# Adult-Onset Fluoxetine Treatment Does Not Improve Behavioral Impairments and May Have Adverse Effects on the Ts65Dn Mouse Model of Down Syndrome

**DOI:** 10.1155/2012/467251

**Published:** 2012-07-16

**Authors:** Markus Heinen, Moritz M. Hettich, Devon P. Ryan, Susanne Schnell, Katharina Paesler, Dan Ehninger

**Affiliations:** DZNE, German Center for Neurodegenerative Diseases, Ludwig-Erhard-Allee 2, 53175 Bonn, Germany

## Abstract

Down syndrome is caused by triplication of chromosome 21 and is associated with neurocognitive phenotypes ranging from severe intellectual disability to various patterns of more selective neuropsychological deficits, including memory impairments. In the Ts65Dn mouse model of Down syndrome, excessive GABAergic neurotransmission results in local over-inhibition of hippocampal circuits, which dampens hippocampal synaptic plasticity and contributes to cognitive impairments. Treatments with several GABA_A_ receptor antagonists result in increased plasticity and improved memory deficits in Ts65Dn mice. These GABA_A_ receptor antagonists are, however, not suitable for clinical applications. The selective serotonin reuptake inhibitor fluoxetine, in contrast, is a widely prescribed antidepressant that can also enhance plasticity in the adult rodent brain by lowering GABAergic inhibition. For these reasons, we wondered if an adult-onset 4-week oral fluoxetine treatment restores spatial learning and memory impairments in Ts65Dn mice. Fluoxetine did not measurably improve behavioral impairments of Ts65Dn mice. On the contrary, we observed seizures and mortality in fluoxetine-treated Ts65Dn mice, raising the possibility of a drug × genotype interaction with respect to these adverse treatment outcomes. Future studies should re-address this in larger animal cohorts and determine if fluoxetine treatment is associated with adverse treatment effects in individuals with Down syndrome.

## 1. Introduction

Down syndrome is caused by trisomy 21 and is frequently associated with cognitive impairments. Based on the partial triplication of chromosome 16, the mouse homologue of human chromosome 21, a mouse model (Ts65Dn) has been developed that shows behavioral abnormalities, including deficient hippocampus-dependent learning and memory [1, 2]. Although most studies regarding the neurobiology of Down syndrome have been focused on neurodevelopment, recent evidence suggests that pathophysiological processes in the adult brain significantly contribute to cognitive impairments in this disorder [3–8]. In Ts65Dn mice, enhanced inhibitory synaptic transmission suppresses proper induction of hippocampal synaptic plasticity, an important cellular mechanism for learning and memory formation [6].

Strikingly, using a variety of different GABA_A_ receptor antagonists to suppress the abnormally increased level of inhibition in adult Ts65Dn mice fully restored their learning and memory impairments without affecting wild-type controls [4]. These beneficial effects were not evident, however, with only acute administration of GABA_A_ receptor antagonists but instead became clear only with a more prolonged (2-3 weeks) treatment [4]. This prolonged treatment was then sufficient to cause improvements in cognitive function lasting well beyond the actual treatment period [4], suggesting that treatment triggered lasting neurobiological changes. Such outcomes are reminiscent of those seen in typical antidepressant treatment of depressed individuals.

The neurobiological basis of these nonacute GABA_A_ receptor antagonist treatment-induced behavioral modifications is currently unknown. Key insights into potential mechanistic aspects, however, may be provided by a brief review of brain development. During postnatal brain development, inhibition plays an important role in regulating the temporal extent of critical periods (i.e., developmental time windows during which sensory input can substantially shape brain structure and function) [9, 10]. The gradually increasing levels of cortical inhibition cause the closing of these critical periods. The mature brain is therefore no longer endowed with such high levels of plasticity. Remarkably, several experimental manipulations that lower inhibitory synaptic transmission have been found to reinstate high levels of plasticity in the adult brain resembling those found in critical periods [11–13]. It is possible that high levels of plasticity, characteristic of critical periods, are actively suppressed in adults by mechanisms including increased inhibition [14]. Therefore, one strategy to enhance plasticity in the adult brain could be the removal of these constraints by lowering inhibition [14, 15].

One pharmacological manipulation particularly interesting from a translational point of view reported that ocular dominance plasticity is reinstated in the mature rat visual cortex by chronic treatment with the widely prescribed antidepressant fluoxetine, presumably also via a decrease in inhibitory synaptic transmission [11]. Vetencourt et al. used brain in vivo microdialysis to show reduced levels of extracellular GABA in the visual cortex of fluoxetine treated animals [11]. White matter LTP, a form of synaptic plasticity that is normally absent in the adult brain due to matured intracortical inhibition, was present in fluoxetine-treated animals [11]. BDNF expression was increased as a consequence of fluoxetine treatment, and intracortical BDNF administration was sufficient to cause an ocular dominance shift in response to monocular deprivation [11]. To test if reduced inhibition underlies the effects of fluoxetine on ocular dominance plasticity, the GABA_A_ receptor agonist diazepam was administered intracortically in fluoxetine-treated mice, which fully occluded the effect of fluoxetine on ocular dominance plasticity [11].

Translation of the preclinical GABA_A_ receptor antagonist findings in Ts65Dn mice (see above) [4, 8] to clinical populations is hampered by the fact that none of the employed GABA_A_ receptor antagonists is currently in clinical use. GABA_A_ receptor antagonists have narrow therapeutic windows and harsh side effect profiles and therefore have limited potential for translational applications, warranting the search for novel treatment approaches. We note that other strategies safer than GABA_A_ receptor antagonists have been proposed for the treatment of Down's syndrome-related cognitive impairments, including GABA_A_ receptor *α*5-selective inverse agonists and other compounds [16–19].

Here, we followed up on the finding that chronic fluoxetine treatment induced reduced levels of inhibition and enhanced plasticity [11], suggesting that chronic fluoxetine administration may have beneficial effects on cognitive dysfunction in Ts65Dn mice, similar to GABA_A_ receptor antagonists. Treatments with selective serotonin reuptake inhibitors (SSRIs), such as fluoxetine, influence the serotonin system and also have complex effects on GABAergic neurotransmission [20–23], including pre- and postsynaptic effects, which may include an inhibition of evoked inhibitory postsynaptic potentials due to elevated extracellular serotonin levels [23]. In the hippocampus, the serotonergic system has been proposed to be involved in shifting inhibition from dendritic to perisomal areas, which should allow for greater dendritic excitation and synaptic plasticity [22].

We tested if an oral fluoxetine treatment regime has therapeutic effects on cognitive impairments (spatial learning in the Morris water maze) in the Ts65Dn mouse model of Down syndrome. There were no beneficial effects of fluoxetine treatment on behavioral impairments in Ts65Dn mice but, instead, unexpected side effects including seizures and death due to treatment that appeared to be genotype specific.

## 2. Material and Methods

### 2.1. Mice

 Experimental animals were generated by crossing C57BL/eJEiJ × C3Sn.BLiA-*Pde6b* + F1 hybrid wild-type males with B6EiC3Sn.BLiA-Ts(17^16^)65Dn/DnJ females (breeders were purchased from The Jackson Laboratories). A 1-1 mating scheme was used, and males were left in the mating cage with the female and her litter. Pups were weaned and tail biopsies for genotyping were obtained at postnatal day 21. Genotyping was performed by qPCR. Experimental animals were housed in groups of 2–4 mice per cage. We kept animals on a 12-hour light-dark cycle. Mice received water and food *ad libitum*. Experiments were carried out during the light period of the cycle. Male and female animals used for experiments were between 149 and 227 days of age at completion of the study (sex and age were approximately balanced across groups). All experiments were performed blinded to genotype and treatment. Local and federal regulations regarding animal welfare were followed.

### 2.2. Pharmacology

 Fluoxetine hydrochloride (Sigma) was administered to the animals through the drinking water at a concentration of 0.2 mg/mL as previously described [11]. Animals were treated for 4 weeks before behavioral assessment commenced and were sacrificed after 6 weeks of treatment.

### 2.3. Water Maze

 Initially, mice were handled for 7 days (for approximately 2 min/animal/day) to habituate the animals to investigator contact and procedural elements associated with the task. Following handling, mice were trained on the hidden version of the water maze. During water maze training, the escape platform was hidden underneath the water surface in a constant location of the pool. The pool (Med Associates) had a diameter of 1.2 m and was filled with opaque water (temperature: 22–24°C). Behavior of the animals was recorded using an automated tracking system (Ethovision XT, Noldus). During training trials, mice were placed into the pool from one of seven randomly assigned starting positions. Each mouse received four daily training trials for 5 consecutive days. Training trials were given in blocks of 2 consecutive trials. Accordingly, intertrial intervals were approximately 1 min between trials 1 and 2, as well as trials 3 and 4, and were approximately 90 min between trials 2 and 3. Training trials were completed when mice climbed on the escape platform or when 1 min had elapsed, whichever came first. Animals were given 15 s posttrial interval on the escape platform after completion of training trials. To evaluate the accuracy with which the animals had learned the position of the escape platform, we performed a probe trial once training was completed. During the probe trial, we removed the escape platform from the pool, and animals were released into the pool from the starting position within the opposite quadrant (OQ). We determined the time that mice spent searching in the target quadrant (which previously contained the escape platform) or the other quadrants during the probe trial. Additionally, we analyzed the number of crossings of the exact target location (i.e., where the platform was during training) and compared it to crossings of analogous positions in the other quadrants. As an additional probe trial measure, we determined the average distance (proximity) to the target location and compared it to the average distance to corresponding locations in the other quadrants. To assess if the probe trial measures differed across genotypes and/or treatment groups, we performed a three-way ANOVA with genotype and treatment as between-subjects factors and quadrant as a within-subject factor. Additionally, to assess for spatial selectivity of searching during the probe trial, we performed *t*-tests to compare target quadrant measures to the corresponding average values of the other quadrants. Escape latencies during training were analyzed by two-way ANOVA with genotype and treatment as between-subjects factors. Swim speed during training and probe trial was analyzed by two-way ANOVA with genotype and treatment as between-subjects factors. Also, distance travelled during the probe trial was analyzed by two-way ANOVA with genotype and treatment as between-subjects factors.

### 2.4. Open Field

Mice were placed for 10 min in a square open field made of acrylic (footprint 27.5 cm × 27.5 cm); activity was recorded and analyzed using an automated system (Ethovision XT, Noldus). Light levels were set to 100 lux in the center of the open field.

### 2.5. Tissue Preparation

 Mice were anesthetized and perfused transcardially with 0.9% saline and 4% paraformaldehyde in cold 0.1 M phosphate buffer (pH 7.4). Brains were extracted, postfixed in 4% paraformaldehyde over night, and subsequently transferred into 30% sucrose. Forty micrometer coronal section series were then created using a sliding microtome (Leica). Sections were stored in cryoprotectant solution (25% ethylene glycol, 25% glycerine, and 0.05 M phosphate buffer) at −20°C.

### 2.6. Immunohistochemistry

 Every sixth section of the coronal section series mentioned above (i.e., sections 240 *μ*m apart) was used for free-floating immunohistochemistry. Sections were rinsed in TBS, incubated in a solution containing 0.6% H_2_O_2_ in TBS to inhibit residual endogenous peroxidase, and blocked for 30 min in TBS with 3% donkey serum and 0.1% Triton X-100. Sections were then incubated for 48 h at 4°C in a primary antibody solution (rabbit anti-choline acetyltransferase (anti-ChAT), Millipore, 1 : 100) containing TBS, 3% donkey serum, and 0.1% Triton X-100. Subsequently, sections were rinsed in TBS, blocked in TBS with 3% donkey serum and 0.1% Triton X-100, and incubated for 1 h at room temperature in secondary antibody (biotinylated donkey anti-rabbit IgG, Dianova, 1 : 500). Next, sections were washed with TBS and subjected to 1 h incubation in avitin-biotin peroxidase complex in TBS (ABC Elite, Vector). Finally, sections were developed with diaminobenzidine (DAB, Roche). Sections were mounted, air-dried, dehydrated with a graded series of ethanol, and mounted with Permount.

### 2.7. Stereology and Image Analysis

Stereological analyses of cell number and cell size of choline acetyltransferase-immunostained cells were performed on section series stained against choline acetyltransferase with sections 240 *μ*m apart, covering the entire fronto-occipital extension of the hemisphere. Analyses were carried out using a Nikon Eclipse 90i microscope equipped with Stereo Investigator (MicroBrightField). Quantitative analysis of the total number of ChAT-positive neurons in the basal forebrain including ventral diagonal band nuclei (VDB) and medial septum nuclei (MSN) was performed in an unbiased fashion using the optical fractionator method as described previously [24, 25]. This method allows for a systematic random sampling of the region of interest (ROI). The landmarks outlining the ROI (MSN and VDB) were taken from the Mouse Brain Atlas [26]. ROIs were manually marked, and pictures from every ROI were taken using the Stereo Investigator software. With a sampling grid of 90 *μ*m × 90 *μ*m systematically moving through the outlined ROI, we counted ChAT-positive neurons that were either within the dissector counting frame (60 *μ*m × 60 *μ*m) or that were touching its right/upper edge. Cell size of ChAT-immunoreactive neurons was determined on the same section series using the nucleator probe within the Stereo Investigator software. Six rays extending from the nucleus were used to mark the boundaries of the cells, allowing an estimation of cell surface area.

## 3. Results

To test if adult-onset fluoxetine treatment has a beneficial effect on behavioral features associated with the Ts65Dn mouse model of Down syndrome, we initiated fluoxetine or vehicle control treatment in Ts65Dn mice and 2N (diploid) littermate controls (2N/vehicle:  *n* = 9  mice; 2N/fluoxetine:  *n* = 11  mice; Ts65Dn/vehicle:  *n* = 7  mice; Ts65Dn/fluoxetine:  *n* = 9  mice).

**Figure 1:**
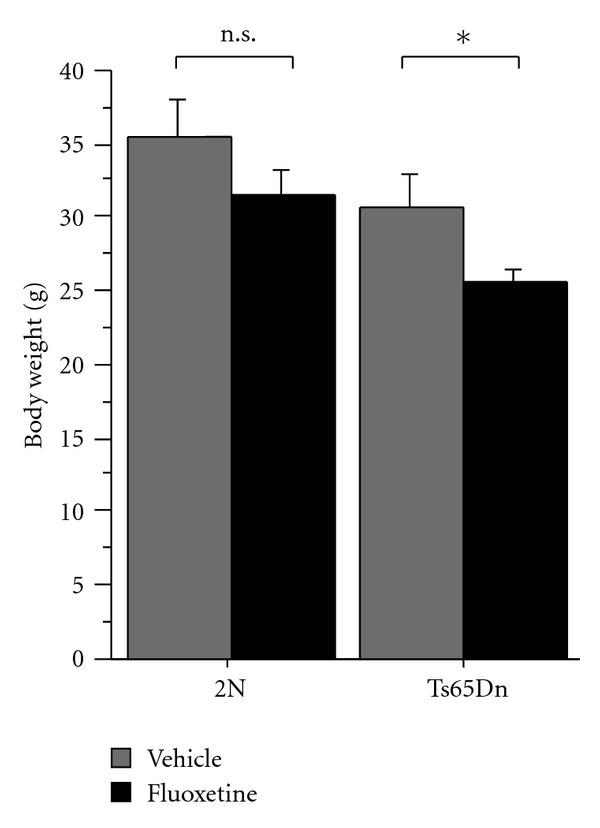
Body weights of Ts65Dn mice and wild-type littermate controls treated with vehicle or fluoxetine (2N, vehicle:  *n* = 8 mice; 2N, fluoxetine:  *n* = 11 mice; Ts65Dn, vehicle:  *n* = 7 mice; Ts65Dn, fluoxetine:  *n* = 7 mice). Two-way ANOVA with genotype and treatment as between-subjects factors showed significant main effects of genotype (ANOVA genotype *F*(1,29) = 7.37,  *P* = 0.01) and treatment (ANOVA treatment *F*(1,29) = 5.42,  *P* = 0.03). Posthoc analyses showed significantly lower body weights in fluoxetine-treated Ts65Dn mice compared to vehicle-treated Ts65Dn mice (Fisher's PLSD,  *P* = 0.04). Body weights in fluoxetine-treated 2N controls were not significantly different from 2N vehicle controls (Fisher's PLSD,  *P* = 0.19). Graph shows mean ± SEM. *denotes comparisons where  *P* < 0.05.  n.s. denotes comparisons where  *P* > 0.05.

**Figure 2:**
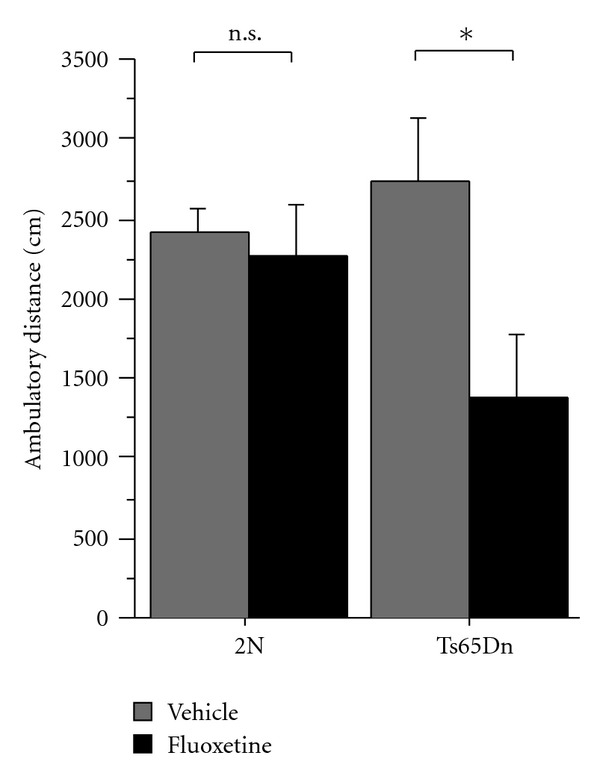
The figure shows the total ambulatory distance in the open field of Ts65Dn mice and wild-type controls treated with either fluoxetine or vehicle (2N, vehicle:  *n* = 8  mice; 2N, fluoxetine:  *n* = 11  mice; Ts65Dn, vehicle:  *n* = 7  mice; Ts65Dn, fluoxetine:  *n* = 7  mice). Two-way ANOVA with genotype and treatment as between-subjects factors revealed a significant effect of drug treatment on ambulatory distance (*F*(1,29) = 5.24,  *P* = 0.03) and suggested a possible genotype × treatment interaction (*F*(1,29) = 3.35,  *P* = 0.08). Posthoc comparison showed significantly reduced ambulatory distance in fluoxetine-treated Ts65Dn mice relative to vehicle-treated Ts65Dn animals (Fisher's PLSD,  *P* = 0.04). In contrast, fluoxetine had no significant effect on ambulatory distance in 2N controls (Fisher's PLSD 2N/fluoxetine versus 2N/vehicle,  *P* = 0.5). The graph shows means ± SEM. *denotes comparisons where  *P* < 0.05.  n.s. denotes comparisons where  *P* > 0.05.

Body weight measurements were taken after 1 month of treatment with fluoxetine or vehicle control (Figure 1; 2N, vehicle:  *n* = 8  mice; 2N, fluoxetine:  *n* = 11  mice; Ts65Dn, vehicle:  *n* = 7  mice; Ts65Dn, fluoxetine:  *n* = 7  mice). Two-way ANOVA with genotype and treatment as between-subjects factors revealed significant main effects of genotype (ANOVA genotype *F*(1,29) = 7.37,  *P* = 0.01) and treatment (ANOVA treatment *F*(1,29) = 5.42,  *P* = 0.03), while there was no significant interaction between the factors (*F*(1,29) = 0.85,  *P* = 0.77). Fluoxetine treatment reduced body weights in Ts65Dn mice relative to Ts65Dn vehicle controls (Fisher's PLSD Ts65Dn/fluoxetine versus Ts65Dn/vehicle,  *P* = 0.04). Fluoxetine had no significant effect on body weight within 2N littermate controls (Fisher's PLSD 2N/vehicle versus 2N/fluoxetine,  *P* = 0.19).

Unexpectedly, during the approximate 6-week treatment period, 4 out of 9 Ts65Dn mice treated with fluoxetine died (Table 1). There were no deaths among the vehicle-treated Ts65Dn mice (0 out of 7 mice) and the fluoxetine-treated wild-type animals (0 out of 11 mice). In the vehicle-treated wild-type group, 1 out of 8 mice was found dead during the treatment period (Fisher's exact test, Ts65Dn/fluoxetine group versus collapsed data from the other groups,  *P* = 0.01).

We started our behavioral assessment of fluoxetine-treated and vehicle-treated Ts65Dn mice and their respective wild-type control groups by testing general exploratory activity in an open field assay (Figure 2; 2N, vehicle:  *n* = 8  mice; 2N, fluoxetine:  *n* = 11  mice; Ts65Dn, vehicle:  *n* = 7  mice; Ts65Dn, fluoxetine:  *n* = 7  mice). Animals were placed into a novel environment, and activity levels were recorded. There was no effect of genotype on total distance travelled in the open field (two-way ANOVA with genotype and treatment as between-subjects factors: *F*(1,29) = 0.73,  *P* = 0.4). Statistical analysis showed a significant effect of drug treatment on activity levels (two-way ANOVA with genotype and treatment as between-subjects factors: *F*(1,29) = 5.24,  *P* = 0.03) and suggested a possible genotype × treatment interaction (two-way ANOVA with genotype and treatment as between-subjects factors: *F*(1,29) = 3.35,  *P* = 0.08). Activity levels were significantly reduced in fluoxetine-treated Ts65Dn mice relative to vehicle-treated trisomic mice (Fisher's PLSD,  *P* = 0.04). Corresponding post hoc analysis did not show a significant effect of treatment in wild-type animals (Fisher's PLSD,  *P* = 0.5).

**Figure 3:**
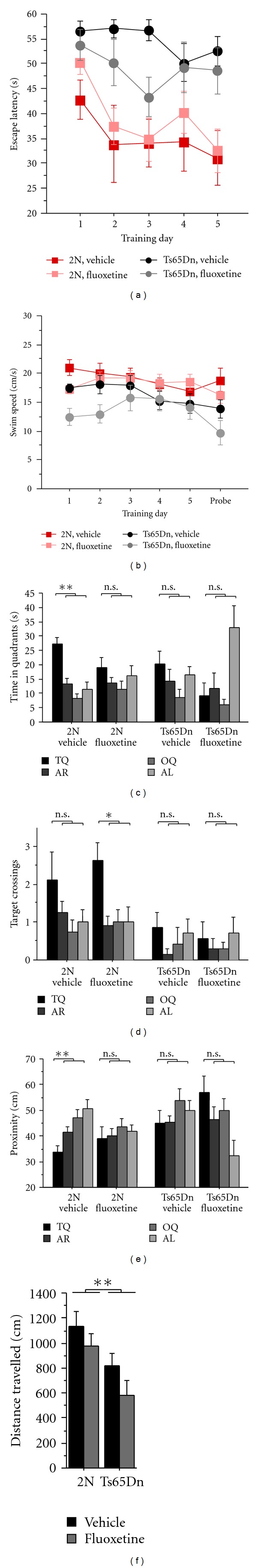
Results from the assessment of Ts65Dn mice and 2N controls in the hidden version of the Morris water maze (2N, vehicle: *n* = 8 mice; 2N, fluoxetine: *n* = 11 mice; Ts65Dn, vehicle: *n* = 7 mice; Ts65Dn, fluoxetine: *n* = 7 mice). (a) Escape latencies. (b) Swim speed during training trials and the probe trial. (c)–(e) Probe trial measures: (c) quadrant occupancy, (d) target crossings, (e) proximity to target, (f) total distance travelled. Pool quadrants: target quadrant (TQ), adjacent right (AR), opposite quadrant (OQ) and adjacent left (AL). Graph shows means ± SEM. **P < 0.01, *P < 0.05, n.s. *P* > 0.05. For details regarding statistical analyses, please see main text.

To determine if fluoxetine treatment restores spatial learning and memory deficits in Ts65Dn mice, we tested Ts65Dn mice and wild-type littermate controls, either treated with fluoxetine or vehicle, in the hidden platform version of the Morris water maze (Figure 3; 2N, vehicle:  *n* = 8  mice; 2N, fluoxetine:  *n* = 11  mice; Ts65Dn, vehicle:  *n* = 7  mice; Ts65Dn, fluoxetine:  *n* = 7  mice). Animals were given 4 daily training trials for 5 consecutive days. Escape latencies decreased during training in all groups (Figure 3(a)), although to a more limited extent in Ts65Dn mice (both vehicle and fluoxetine treated). Statistical analysis revealed a significant main effect of genotype with higher escape latencies in Ts65Dn mice compared to 2N controls (two-way ANOVA with genotype and treatment as between-subjects factors: *F*(1,29) = 20.11,  *P* = 0.0001). There was no significant main effect of treatment (two-way ANOVA: *F*(1,29) = 0.07,  *P* = 0.79) and no genotype × treatment interaction (two-way-ANOVA: *F*(1,29) = 2.07,  *P* = 0.16). We analyzed swim speed during all training sessions and the probe trial using repeated-measures two-way ANOVA with genotype and treatment as between-subjects factors (Figure 3(b)). Swim speed was significantly decreased in Ts65Dn mice relative to 2N controls (repeated-measures two-way ANOVA: *F*(1,29) = 9.15,  *P* = 0.005). There was no significant effect of treatment on swim speed (repeated-measures two-way ANOVA: *F*(1,29) = 2.258,  *P* = 0.144) and no significant genotype × treatment interaction (repeated-measures two-way ANOVA: *F*(1,29) = 0.529,  *P* = 0.473). 

To evaluate how accurately the mice had learned the escape platform position during training, we performed a single-probe trial (no platform in the water tank) after completion of training (Figures 3(c), 3(d), and 3(e)). We analyzed several probe trial measures that report spatial selectivity of searching and, hence, indicate the extent of spatial learning that occurred.

First, we measured the time that the animals spent in the target quadrant (which previously contained the escape platform) and the other quadrants during the probe trial (Figure 3(c)). Statistical analysis of the quadrant occupancy data showed significant genotype × quadrant and treatment × quadrant interactions (three-way ANOVA with genotype and treatment as between-subjects factors and quadrant as within-subjects factor: genotype × quadrant interaction, *F*(3,116) = 5.24;  *P* = 0.002, treatment × quadrant interaction: *F*(3,116) = 5.48,  *P* = 0.002), indicating that genotype and treatment had significant effects on quadrant occupancy. Vehicle-treated 2N controls spent significantly more time in the target quadrant than the other quadrants (*t*-test, target quadrant occupancy versus average occupancy of the other quadrants:  *P* = 0.001), indicating memory for the platform location. In contrast, in the other groups, target quadrant occupancy was not significantly different from average occupancy of the other quadrants (*t*-test, target quadrant occupancy versus average occupancy of the other quadrants: 2N/fluoxetine,  *P* = 0.292; Ts65Dn/vehicle,  *P* = 0.267; Ts65Dn/fluoxetine,  *P* = 0.241).

We also recorded the number of target crossings (i.e., crossings of the exact target location, where the platform was located during training) during the probe trial, which was compared to the number of crossings over corresponding locations in the other quadrants (Figure 3(d)). With respect to target crossings, ANOVA analyses yielded a significant effect of genotype (three-way ANOVA with genotype and treatment as between-subjects factors and quadrant as within-subjects factor: *F*(1,116) = 17.3,  *P* < 0.0001), reflecting an overall reduced number of crossings in Ts65Dn mice and, additionally, a possible genotype × quadrant interaction (three-way ANOVA with genotype and treatment as between-subjects factors and quadrant as within-subjects factor: *F*(3,116) = 2.25,  *P* = 0.09). To further probe for spatial selectivity of searching, we asked whether animals showed significantly more crossings over the target location than over corresponding locations in the other quadrants. Fluoxetine-treated 2N mice showed significantly more crossings of the target location than the other locations (*t*-test, target crossings versus average crossings of corresponding locations in the other quadrants:  *P* = 0.010), while this was not the case for the other groups (*t*-test, target crossings versus average crossings of corresponding locations in the other quadrants: 2N/vehicle,  *P* = 0.100; Ts65Dn/vehicle,  *P* = 0.362; Ts65Dn/fluoxetine,  *P* = 0.784).

As a third measure for spatial selectivity of searching, we looked at proximity to the target (i.e., average distance to the target location, where the platform was located during training) and compared it to the average distance to corresponding locations in the other quadrants (Figure 3(e)). Three-way ANOVA with genotype and treatment as between-subjects factors and quadrant as within-subjects factor showed a significant main effect of genotype (*F*(1,116) = 7.10,  *P* = 0.009), which was reflective of the overall larger distances that Ts65Dn mice had to the target position and corresponding positions in the other quadrants. This ANOVA also revealed a significant genotype × quadrant interaction (*F*(3,116) = 4.10,  *P* = 0.008) and a significant treatment × quadrant interaction (*F*(3,116) = 5.25,  *P* = 0.002), showing that the search pattern was influenced by genotype and treatment, such that target-preferential searching was less pronounced or absent in Ts65Dn mice/fluoxetine-treated mice. Vehicle-treated 2N mice showed significantly lower proximity values to the target than average distance to the other positions (*t*-test, proximity to target versus average proximity to corresponding locations in the other quadrants:  *P* = 0.005), again indicating preferential searching in the relative vicinity of the target in this group. This comparison yielded nonsignificant results for the other groups (*t*-test, target crossings versus average crossings of corresponding locations in the other quadrants: 2N/fluoxetine,  *P* = 0.550; Ts65Dn/vehicle,  *P* = 0.442; Ts65Dn/fluoxetine,  *P* = 0.077). As expected based on the slower swim speed in Ts65Dn mice (see above), total distance travelled during the probe trial was significantly reduced in Ts65Dn mice (Figure 3(f); two-way ANOVA with genotype and treatment as between-subjects factors: genotype effect, *F*(1,29) = 10.98,  *P* = 0.003; treatment effect, *F*(1,29) = 3.21,  *P* = 0.084, genotype × treatment interaction, *F*(1,29) = 0.14,  *P* = 0.706).

Taken together, the probe trial revealed indications of spatially selective searching and, hence, successful spatial learning and memory in vehicle-treated (see quadrant occupancy and proximity to target) and fluoxetine-treated (see target crossings) 2N mice, but not in Ts65Dn mice, irrespective of treatment group. These data show clear behavioral abnormalities of Ts65Dn mice in the water maze, which are consistent with published data [2, 8, 27] and reflect motor impairments (see swim speed) and spatial learning and memory difficulties (see probe trial measures) in Ts65Dn mice. Neither motor impairments nor spatial learning deficits in Ts65Dn mice were improved by fluoxetine treatment.

During behavioral experimentation, we incidentally witnessed tonic-clonic seizures triggered by handling in a few animals (a total of 3 mice) (Table 2). All seizure episodes observed occurred in Ts65Dn mice on fluoxetine treatment. Seizures were not observed in the other groups (Fisher's exact test, Ts65Dn/fluoxetine group versus collapsed data from the other groups,  *P* = 0.01).

**Figure 4:**
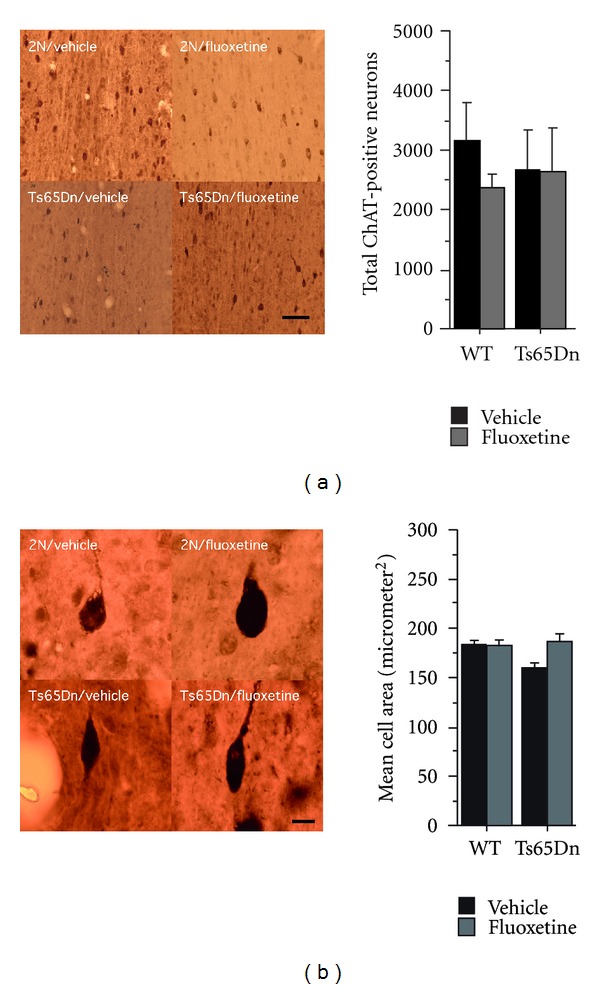
Results from stereological assessment of choline acetyltransferase-(ChAT-) positive cells in basal forebrain cholinergic nuclei in Ts65Dn mice and wild-type controls treated either with fluoxetine or vehicle control (2N, vehicle:  *n* = 8  mice; 2N, fluoxetine:  *n* = 11  mice; Ts65Dn, vehicle:  *n* = 7  mice; Ts65Dn, fluoxetine: *n* = 5 mice). (a) Stereological counting of choline acetyltransferase-(ChAT-) positive cells in basal forebrain cholinergic nuclei. Shown are representative brain sections from 2N controls and Ts65Dn mice under treatment with vehicle or fluoxetine. Scale bar = 50 *μ*m. The quantification is shown in the bar graph. No differences with regards to cholinergic cell number were detectable between Ts65Dn mice and 2N controls (two-way ANOVA with genotype and treatment as between-subjects factors: genotype effect, *F*(1,27) = 0.04,  *P* = 0.840; treatment effect, *F*(1,27) = 0.61,  *P* = 0.441; genotype × treatment interaction, *F*(1,27) = 0.49,  *P* = 0.491). Graph shows means ± SEM. (b) Cell size of choline acetyltransferase-(ChAT-) positive cells in basal forebrain cholinergic nuclei from Ts65Dn mice and 2N controls. Shown are representative ChAT-stained neurons from euploid mice treated with vehicle or fluoxetine and Ts65Dn mice treated with vehicle or fluoxetine. The bar graph is depicting cell size distributions of ChAT-immunoreactive cells. Cholinergic neurons of euploid mice appeared to be larger than in Ts65Dn mice although the statistical comparison was not significant (two-way ANOVA with genotype and treatment as between-subjects factors: genotype effect, *F*(1,27) = 2.2,  *P* = 0.15). Fluoxetine treatment had no significant effect on cell size, although there was a trend towards increasing cell size in Ts65Dn mice (two-way ANOVA with genotype and treatment as between-subjects factors: treatment effect, *F*(1,27) = 3.08,  *P* = 0.091; genotype × treatment interaction, *F*(1,27) = 3.14,  *P* = 0.08). The graph shows means ± SEM.

One of the neuropathological hallmarks of both Down syndrome and Alzheimer's disease is the age-dependent loss of basal forebrain cholinergic neurons (BFCNs) [24, 28]. Previous studies pointed to a correlation between BFCN degeneration and abnormalities in memory and learning in aging Ts65Dn mice [24]. To probe whether fluoxetine is able to ameliorate or prevent early BFCN degeneration in Ts65Dn mice, we performed unbiased stereological cell counts of choline acetyltransferase (ChAT) antibody-stained sections in fluoxetine-treated animals and vehicle controls. We determined the cholinergic cell number and size (Figure 4) in the medial septal nuclei (MSN) and ventral diagonal band (VDB) of each group (2N, vehicle:  *n* = 8 mice; 2N, fluoxetine:  *n* = 11 mice; Ts65Dn, vehicle:  *n* = 7 mice; Ts65Dn, fluoxetine:  *n* = 5 mice).

No clear differences with regards to cholinergic cell number were evident between trisomic and euploid mice (two-way ANOVA with genotype and treatment as between-subjects factors: *F*(1,27) = 0.04,  *P* = 0.84). Treatment also had no obvious effects on the number of ChAT-positive cells in basal forebrain cholinergic nuclei (two-way ANOVA with genotype and treatment as between-subjects factors: *F*(1,27) = 0.61,  *P* = 0.44). Since neuronal atrophy precedes cell loss [28], we analyzed cell size of ChAT-positive cells in BFCN of fluoxetine-/vehicle-treated Ts65Dn mice/wild-type controls. Cholinergic neurons of euploid mice were insignificantly larger than those in trisomic mice (two-way ANOVA with genotype and treatment as between-subjects factors: *F*(1,27) = 2.2,  *P* = 0.15). Fluoxetine treatment had no significant effect on cell size, although there was a trend towards increasing cell size in Ts65Dn mice (two-way ANOVA with genotype and treatment as between-subjects factors: treatment effect, *F*(1,27) = 3.08,  *P* = 0.091; genotype × treatment interaction, *F*(1,27) = 3.14,  *P* = 0.08).

## 4. Discussion

In the present study, we set out to test whether a 4-week, adult-onset fluoxetine treatment is effective against behavioral alterations in the Ts65Dn mouse model of Down syndrome. We found no discernible benefits of this fluoxetine treatment regimen in Ts65Dn mice. In particular, we did not observe a treatment effect on the clear Ts65Dn behavioral phenotype in the water maze, which included slower swim speed and effects on probe trial measures indicative of spatial learning and memory impairments.

Adverse side effects due to fluoxetine treatment of Ts65Dn mice, however, appeared to be profound in our cohort. Four out of 9 Ts65Dn mice died while on fluoxetine treatment during which time there were no deaths in the other groups (with the exception of 1 dead wild-type/vehicle animal). These results suggest that fluoxetine may have a genotype-specific adverse effect and warrant further experimentation in larger animal cohorts that also assess the cause of death in fluoxetine-treated Ts65Dn mice.

We observed handling-induced seizures in a few mice, all of which were fluoxetine-treated Ts65Dn mice, suggesting interactive effects of Ts65Dn genotype and fluoxetine treatment on seizure susceptibility. Seizures represent a relatively rare side effect of therapeutic fluoxetine regimens in general clinical populations but are a more common feature of fluoxetine intoxications [29–31]. Several epilepsy paradigms in animal models also illustrate a proconvulsive effect of fluoxetine; for instance, fluoxetine pretreatment potentiates the convulsive effects of pentylenetetrazol (PTZ) and electrically evoked seizures [32, 33]. Collectively, these reports show that treatments with selective serotonin reuptake inhibitors, including fluoxetine, can lower seizure thresholds in humans and animal models.

Epilepsy is not uncommon in Down syndrome and may affect 1–13% of all individuals with trisomy 21 [34, 35]. Although spontaneous seizures are not a feature of the Ts65Dn model, Ts65Dn mice are more prone to audiogenic seizures than controls [36], suggesting reduced seizure thresholds in this model. In sum, the data raise the possibility that behavioral convulsions in Ts65Dn mice, described above, may result from an interaction between fluoxetine and the lowered seizure thresholds in Ts65Dn mice. Future studies should examine these effects in larger animal cohorts and include electrophysiological assessments of seizure activity.

For the present study, we adopted a fluoxetine treatment regime used previously [11], that is, administration of 0.2 mg/mL fluoxetine via the drinking water. This treatment regime was found to lower intracortical inhibition and to reinstate ocular dominance plasticity in the adult visual cortex [11]. The fluoxetine dose is higher than rodent equivalents of clinically used doses (i.e., clinically employed doses converted into equivalent surface area doses for mice/rats) but is within the range of fluoxetine dosing schemes generally used in mice and rats (e.g., [20, 37, 38]). In future work, it will be important to establish a dose-response function with respect to the side effects in Ts65Dn mice. Based on our present data, we cannot rule out the possibility that lower fluoxetine doses exert beneficial effects in the Ts65Dn model of Down syndrome. It will also be important to determine if drug × genotype interactions are caused by genotype-dependent differences in CNS effects of fluoxetine or, possibly, different pharmacokinetic profiles in Ts65Dn mice and wild-type controls (e.g., metabolism of fluoxetine, tissue distribution, etc.).

The animals used in the present study were partly in the age range in which neurodegenerative processes may slowly begin in Ts65Dn mice (i.e., >6 months of age). We assessed neurodegeneration via stereological quantification of basal forebrain cholinergic neurons and did not observe significant differences between trisomic and euploid animals (Figure 4), showing that neurodegenerative changes were not a prominent feature in our Ts65Dn cohort (consistent with other reports showing that first signs of degenerative changes affecting the cholinergic system in Ts65Dn mice emerge between 6 and 12 months; [28, 39]). Nevertheless, we cannot rule out the possibility that subtle degenerative changes were already present, yet not measureable in our cohort. Therefore, it remains possible that adult-onset fluoxetine treatment has beneficial effects in 3–6 months old Ts65Dn mice (i.e., in the narrow time window during adulthood, wherein Ts65Dn mice are thought to be free of degenerative changes).

A prior study had assessed the effect of fluoxetine treatment on hippocampal neurogenesis in 2–5 months old Ts65Dn mice [40]. Fluoxetine was administered i.p. and treatment was shorter (15 days) and involved lower dosing (5 mg/kg) than treatment in the present study. Fluoxetine treatment increased neurogenesis in Ts65Dn mice and wildtype controls [40], which is in agreement with earlier reports regarding the effects of chronic fluoxetine treatment on adult hippocampal neurogenesis [41]. Behavioral results were not reported and, hence, it remains unclear whether there was a behavioral effect associated with fluoxetine treatment of Ts65Dn mice in this study.

Another more recent study reported beneficial effects of fluoxetine, administered early postnatally, in the Ts65Dn mouse model [42]. In this study, fluoxetine was administered from P3 to P15 (s.c. injections of 5 mg/kg from P3 to P7 and 10 mg/kg from P8 to P15) followed by a BrdU injection at P15 to label proliferating cells. Fluoxetine increased cell proliferation and generation of new neurons in the dentate gyrus and subventricular zone of Ts65Dn mice and wild-type controls. Fluoxetine treatment increased the number of dentate gyrus granule cells at P45 in Ts65Dn mice to wild-type control levels, while granule cell numbers were reduced in vehicle Ts65Dn mice. Limited behavioral assessment was performed in the context of this study in juveniles (at P43). Context fear-conditioning impairments in juvenile Ts65Dn mice were improved by early postnatal fluoxetine treatment, while fluoxetine had no effects on wild-type controls. In the present study we did not find beneficial effects of fluoxetine treatment on behavioral impairments of adult Ts65Dn mice in the Morris water maze. It is possible that early postnatal and adult-onset fluoxetine treatments have different effects on behavioral impairments in Ts65Dn mice. The protocols in our study, however, differ also in a number of other respects from the published paper (e.g., fluoxetine dose, route of administration, and duration of treatment; behavioral paradigm: here, we assessed learning in the water maze; Bianchi et al. assessed fear conditioning), which may also account for the different findings. In future studies, it will therefore be important to further explore this parameter space, that is, to establish dose-response relationships for fluoxetine treatment in Ts65Dn mice at different ages and to conduct comprehensive behavioral assessments that cover impairments of Ts65Dn mice in a range of behavioral tasks.

## 5. Conclusion

Here, we determined the impact of an adult-onset, chronic treatment with the antidepressant fluoxetine on behavioral alterations in the Ts65Dn mouse model of Down syndrome. We did not find a beneficial effect of fluoxetine treatment on Ts65Dn behavioral phenotypes, but instead our findings suggest the presence of genotype-dependent fluoxetine side effects; we observed seizures and mortality in treated Ts65Dn mice, but not wild-type controls. Future studies should reevaluate these findings in larger animal cohorts, determine what the nature of the possible drug × genotype interaction is (e.g., genotype-dependent differences in drug metabolism, tissue distribution, or truly differential effects of equivalent drug tissue concentrations on cellular/tissue functions), and establish dose-response relationships for these possible side effects.

## Figures and Tables

**Table 1:** Number of deaths during treatment with fluoxetine or vehicle in Ts65Dn mice and wild-type controls.

	2N	Ts65Dn
Vehicle	1 (12.5%)	0 (0%)
Fluoxetine	0 (0%)	4 (44.4%)

**Table 2:** Number of animals displaying seizures stratified by genotype and treatment group.

	2N	Ts65Dn
Vehicle	0 (0%)	0 (0%)
Fluoxetine	0 (0%)	3 (33.3%)
